# Immune cell identifier and classifier (ImmunIC) for single cell transcriptomic readouts

**DOI:** 10.1038/s41598-023-39282-4

**Published:** 2023-07-26

**Authors:** Sung Yong Park, Sonia Ter-Saakyan, Gina Faraci, Ha Youn Lee

**Affiliations:** Department of Molecular Microbiology and Immunology, Keck School of Medicine, University of Southern California, Los Angeles, USA

**Keywords:** Computational biology and bioinformatics, Data processing, Software

## Abstract

Single cell RNA sequencing has a central role in immune profiling, identifying specific immune cells as disease markers and suggesting therapeutic target genes of immune cells. Immune cell-type annotation from single cell transcriptomics is in high demand for dissecting complex immune signatures from multicellular blood and organ samples. However, accurate cell type assignment from single-cell RNA sequencing data alone is complicated by a high level of gene expression heterogeneity. Many computational methods have been developed to respond to this challenge, but immune cell annotation accuracy is not highly desirable. We present ImmunIC, a simple and robust tool for immune cell identification and classification by combining marker genes with a machine learning method. With over two million immune cells and half-million non-immune cells from 66 single cell RNA sequencing studies, ImmunIC shows 98% accuracy in the identification of immune cells. ImmunIC outperforms existing immune cell classifiers, categorizing into ten immune cell types with 92% accuracy. We determine peripheral blood mononuclear cell compositions of severe COVID-19 cases and healthy controls using previously published single cell transcriptomic data, permitting the identification of immune cell-type specific differential pathways. Our publicly available tool can maximize the utility of single cell RNA profiling by functioning as a stand-alone bioinformatic cell sorter, advancing cell-type specific immune profiling for the discovery of disease-specific immune signatures and therapeutic targets.

## Introduction

Single cell RNA sequencing is an invaluable tool for immune profiling, providing the transcriptomic landscape of thousands of individual cells. This method has identified specific immune cells as disease markers^1,2^ and suggested potential therapeutic target genes of disease-associated immune cells^3^. The utility of single cell RNA sequencing has also been demonstrated by investigating immune and non-immune cell communication in the tumor microenvironment^4^. Furthermore, this single cell approach has designated immune signatures of disease severity, as reported in recent COVID-19 studies^5,6^. Taken together, single cell RNA sequencing has advanced our understanding of the immune system and aided in drug discovery for diverse diseases.

High-resolution immune profiling requires reliable cell-type classification. However, gene expression patterns within a single immune cell type can be heterogeneous with different study conditions. This heterogeneity hinders consistent cell type annotation from single cell RNA sequencing data. The current approach of unsupervised clustering^7^ is sensitive to these study-specific gene expression profiles. Furthermore, ad hoc annotation for each cluster is a non-standardized step and thus cell assignment outcomes may not be reproducible. Alternative approaches for immune cell-type assignment include Garnett^8^, CellAssign^9^, Cell BLAST^10^, CellTypist^11^, scGate^12^, and scType^13^. However, these methods have not been validated by a large-scale data of diverse immune cells collected from many different studies.

We here present ImmunIC (Immune cell Identifier and Classifier) as an accurate and automated tool for human immune cell classification. To properly address diverse study-specific gene expression signatures, we first compiled 66 independent single cell RNA sequencing studies and collect over two million immune cells and a half million non-immune cells. We took advantage of the predetermined leukocyte gene signature matrix^14^ (LM22) to filter immune cells from a mixture of immune and non-immune cells. Using the maximum correlation approach similar to SingleR^15^, we subsequently categorized the identified immune cells into B cells, plasma cells, T cells, NK cells, monocytes, dendritic cells, macrophages, neutrophils and other myeloid cells. We then added a machine learning method called Xgboost^16^ to further enhance the classification resolution between CD4+ and CD8+ T cells. We extensively benchmarked the accuracy of ImmunIC against the currently available methods. We also demonstrated ImmunIC’s utility by identifying immune cell-type specific differential pathways from previously published peripheral blood mononuclear cell (PBMC) data of severe COVID-19 cases and healthy controls^5^.

## Results

### Single cell RNA sequencing datasets

We compiled publicly available single cell RNA sequence datasets from 66 different studies, as presented in Tables 1 and 2. The total datasets consisted of 2,078,671 immune cells and 509,300 non-immune cells. These data were obtained using different platforms: (i) 10 × Genomics^17^, (ii) Smart-seq2^18^, (iii) MARS-seq^19^, (iv) Seq-Well^20^, (v) Drop-seq^21^ and BD Rhapsody Single-Cell Analysis System^22^. The immune cell group included 999,462 PBMCs from 173 individuals^5^ and 81,713 PBMCs from 20 individuals^23^. The lymphocyte group consists of 332,336 B cells from four different studies^5,17,24,25^, 42,777 plasma cells^26^, 100,411 T cells^5,27^, 173,996 CD4+ T cells^17,28–33^, 156,960 CD8+ T cells^17,30,34–36^, and 28,570 NK cells^17,37,38^. In the immune cell group, there were 23,705 myeloid cells^39–41^, 49,687 monocytes^17,42–46^, 7,739 dendritic cells^43,47,48^, 999 macrophages^49^, and 80,316 neutrophils^46,50^. Immune cell types were determined polychromatic flow cytometry-based immunophenotyping measures in the original publications. Table 1 presents each study’s accession number, source publication and number of cells. The non-immune cell group consists of 22 different cell types from 26 studies, including 14,537 intestine cells^51^, 4,524 kidney cells^52^, 2,249 neuroblastoma cells^53^, and 5,680 breast cancer cells^54^ (Table 2).

**Table 1:** ImmunIC’s accuracy on single cell RNA sequencing datasets of immune cells.

Cell type	Study ID	Study accession number	Number of cells	Classification accuracy
PBMC	PBMC-1 PBMC-2	GSE158055^5^ GSE149689^23^	999,462 81,713	99.3% [99.3–99.3%] 83.4% [83.2–83.7%]
B cells	BC-1 BC-2 BC-3 BC-4 PLASMA	GSE158055^5^ E-MTAB-9005^24^ ^17^ GSE135710^25^ GSE117156^26^	294,643 24,946 10,047 2,700 42,777	98.6% [98.6–98.6%] 91.5% [91.1–91.8% 99.8% [99.7–99.9%] 77.6% [76.0–79.2%] 75.9% [75.5–76.3%]
T cells	TC-1 TC-2	GSE126030^27^ GSE158055^5^	63,861 36,550	81.1% [80.8–81.4%] 73.3% [72.8–73.7%]
CD4+ T cells	CD4-1 CD4-2 CD4-3 CD4-4 CD4-5 CD4-6 CD4-7 CD4-8 CD4-9 CD4-10 CD4-11	EGAS00001003215^28^ GSE121267^29^ ^17^ GSE99254^30^ ^17^ ^17^ ^17^ GSE150132^31^ GSE99254^30^ GSE119373^32^ GSE122846^33^	43,112 3,149 11,180 5,300 10,162 10,427 10,224 73,566 2,098 2,892 1,886	94.1% [93.9–94.3%] 86.2% [85.0–87.4%] 96.2% [95.9–96.6%] 97.0% [96.5–97.4%] 95.6% [95.2–96.0%] 95.3% [94.9–95.7%] 94.5% [94.1–94.9%] 88.3% [88.1–88.6%] 97.5% [96.9–98.2%] 96.8% [96.1–97.4%] 95.4% [94.5% -96.4%]
CD8+ T cells	CD8-1 CD8-2 CD8-3 CD8-4 CD8-5 CD8-6	GSE99254^30^ ^17^ GSE180268^34^ GSE169503^35^ ^17^ GSE159252^36^	4,439 10,192 56,470 4,487 11,915 69,457	88.7% [87.8–89.6%] 90.4% [89.9–91.0%] 93.3% [93.1–93.5%] 82.1% [81.0–83.2%] 90.7% [90.2–91.2%] 96.6% [96.5–96.7%]
NK cells	NK-1 NK-2 NK-3	^17^ GSE130430^37^ GSE144430^38^	8,339 11,036 9,195	98.8% [98.5–99.0%] 96.0% [95.7–96.4%] 96.5% [96.2–96.9%]
Myeloid cells	Myeloid-1 Myeloid-2 Myeloid-3	GSE125680^39^ GSE165907^40^ GSE115007^41^	4,434 11,213 8,058	72.6% [71.3–73.9%] 84.6% [84.0–85.3%] 71.0% [70.0–72.0%]
Monocytes	Mono-1 Mono-2 Mono-3 Mono-4 Mono-5 Mono-6	GSE103544^42^ ^17^ GSE94820^43^ GSE126085^44^ GSE146974^45^ ^46^	856 2,612 372 197 10,878 34,772	96.5% [95.3–97.7%] 82.5% [81.1–84.0%] 89.5% [86.4–92.6%] 70.1% [63.7–76.4%] 99.6% [99.5–99.7%] 98.5% [98.3–98.6%]
Dendritic cells	DC-1 DC-2 DC-3	GSE153835^47^ GSE94820^43^ GSE89232^48^	7,383 192 164	84.7% [83.9–85.6%] 79.7% [74.0–85.4%] 87.2% [82.1–92.3%]
Macrophages	Macro	GSM3633042^49^	999	77.2% [74.6–79.8%]
Neutrophils	Neutro-1 Neutro-2	^46^ GSE137540^50^	61,537 18,779	93.7% [93.5–93.9%] 70.7% [70.0–71.3%]

**Table 2:** Single cell RNA sequencing datasets of non-immune cells.

Cell type	Study accession number	Number of cells	Proportion of cells classified as non-immune cells
Intestine cells	GSE125970^51^	14,537	99.9% [99.8–99.9%]
Kidney cells	GSE118184^52^	4,524	98.2% [97.8–98.6%]
Airway epithelial cells	GSE134174^64^	36,248	99.4% [99.4–99.5%]
Prostate cells	GSE117403^65^	109,061	97.0% [96.9–97.1%]
Oligodendrocytes	GSE118257^66^	16,046	96.3% [96.0–96.6%]
Pancreatic islets cells	GSE114297^67–69^	20,784	99.5% [99.4–99.6%]
Pancreatic islets cells	GSE101207^55^	19,959	99.7% [99.6–99.8%]
Pancreatic islets cells	GSE124742^70^	7,636	98.6% [98.3–98.9%]
Keratinocytes	GSE155817^71^	22,922	99.5% [99.4–99.6%]
Neuroblastoma cells	GSE163431^53^	2,249	100% [100–100%]
Retina cells	GSE137537^72^	12,424	99.4% [99.3–99.6%]
Retina cells	GSE130636^73^	8,217	98.0% [97.7–98.3%]
Fovea retina cells	GSE148077^74^	86,253	99.1% [99.0–99.1%]
Melanoma cells	GSE134432^75^	38,032	99.4% [99.3–99.5%]
Entorhinal cortex cells	GSE138852^76^	11,503	97.5% [97.3–97.8%]
Liver progenitor-like cells	GSE116113^77^	7,459	98.8% [98.6–99.1%]
Mesothelial cells	GSE149583^78^	12,592	100% [100–100%]
Breast epithelial cells	GSE113196^79^	24,646	96.5% [96.3–96.8%]
Neuroendocrine tumor cells	GSE140312^80^	4,738	95.0% [94.4–95.6%]
Oocytes	GSE107746^81^	151	99.3% [98.0–100%]
Fibroblasts	GSE150311^82^	10,986	98.0% [97.7–98.3%]
Oligodendroglioma cells	GSE70630^83^	4,347	94.1% [93.4–94.8%]
Medulloblastoma cells	GSE119926^84^	8,691	100% [100–100%]
Brain tumor cells	^85^	1,615	66.4% [64.1–68.7%]
Breast cancer cells	^54^	5,680	96.9% [96.5–97.4%]
Substantia nigra cells	GSE140231^86^	18,000	97.8% [97.6–98.0%]

### Immune cell identification by ImmunIC

**Figure 1:**
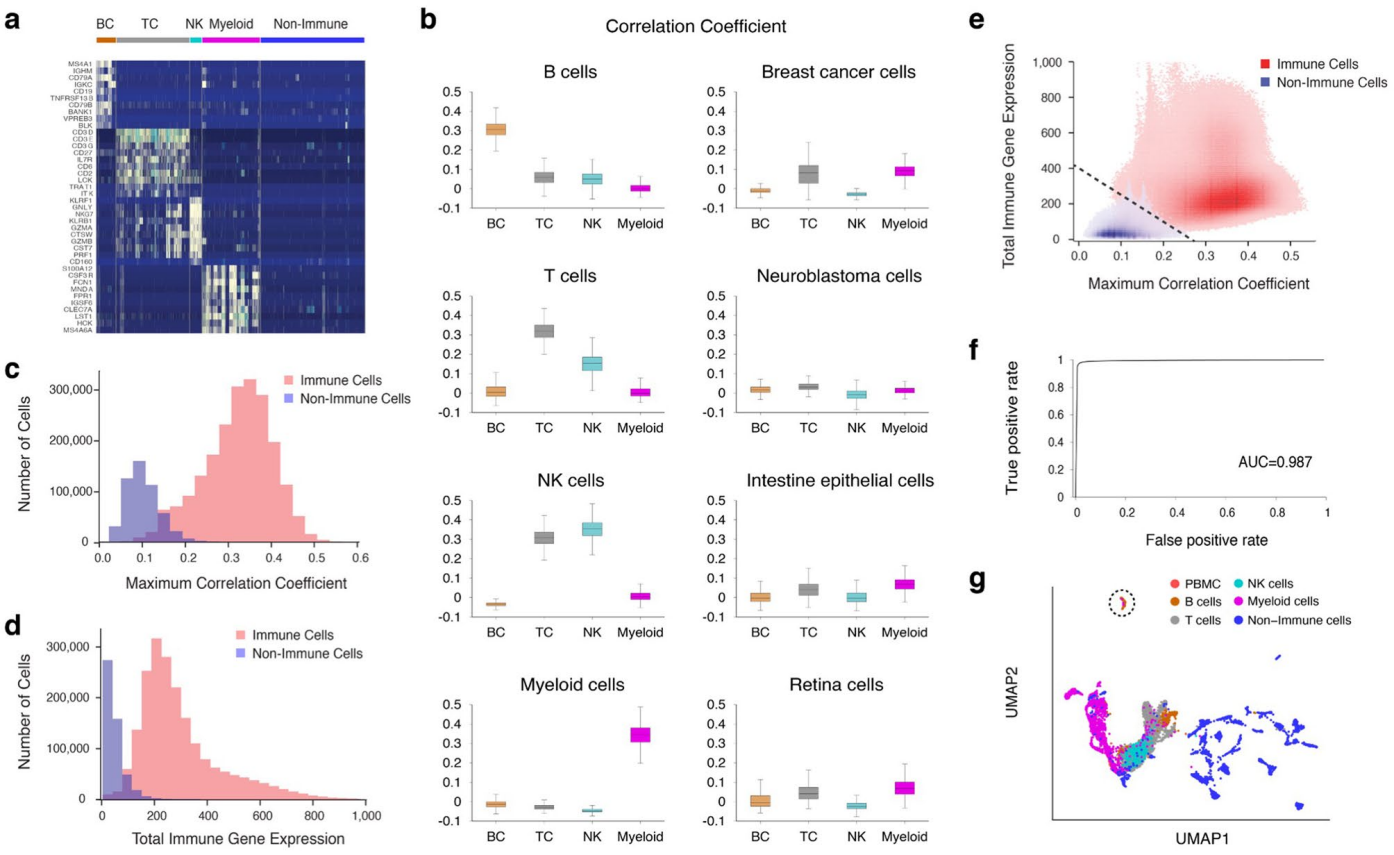
Immune and non-immune cell classification. (**a**) Top 10 upregulated genes from LM22’s 547 genes for B cells (BC), T cells (TC), NK cells (NK), and myeloid cells (Myeloid) (ordered by adjusted p value). Non-immune cells consist of 22 different cell types including intestine cells, kidney cells, neuroblastoma cells and breast cancer cells. A total of 100 cells were randomly selected from each dataset listed in Tables 1 and 2 for the purpose of visualization. (**b**) Pearson’s correlation coefficients of different types of immune cells to LM22’s B cell profile (BC), T cell profile (TC), NK cell profile (NK) and myeloid cell profile (Myeloid). (**c**) Maximum correlation coefficient distributions of 2,078,671 immune cells in Table 1 (red) and 509,300 non-immune cells in Table 2 (blue). (**d**) Total immune gene expression distributions of these immune cells (red) and non-immune cells (blue). (**e**) Separation of these immune cells (red) and non-immune cells (blue) by the dotted line in the maximum correlation coefficient and total immune gene expression plane. (**f**) Binary classification of these immune and non-immune cells with the area under the ROC curve (AUC) of 0.987. (**g**) Unsupervised clustering analysis^56^ on immune and non-immune cells using Seurat 4.0^7^. Immune cells consisting of PBMCs, B cells, T cells, NK cells, myeloid cells and 22 different types of non-immune cells (Tables 1 and 2). Plasma cells are presented in the dotted circle.

We identified immune cells using the Leukocyte signature matrix (LM22) which consists of 547 marker genes^14^, as summarized in ImmunIC’s workflow (see Supplementary Fig. S1). Each immune cell type has a unique gene marker combination in this signature matrix^14^. We observed that many genes in LM22 were uniquely expressed in each cell type at single cell resolution and these genes’ expressions were fairly low in non-immune cells (Fig. 1a and Supplementary Fig. S2). Therefore, we measured the correlation coefficient between each cell’s gene expression and each LM22 profile. B cells showed an average Pearson’s correlation coefficient of 0.31 with LM22’s B cell profile, but had less than 0.1 to T cell, NK cell, and myeloid cell profiles (Fig. 1b). Likewise, T cells, NK cells and myeloid cells showed the greatest correlation coefficient to their own cell types’ profiles (Fig. 1b). However, non-immune cells such as breast cancer cells, neuroblastoma cells, intestine epithelial cells and retina cells showed comparable correlations (< 0.2) with all immune cell types (Fig. 1b). By defining the maximum correlation coefficient as the highest coefficient to LM22, we compared its distribution of around two million immune cells with that of a half-million non-immune cells (Fig. 1c). We observed a clear difference between the immune and non-immune cells, suggesting that the maximum correlation coefficient is a robust metric not only for annotating immune cell type but also for differentiating immune and non-immune cells with single-cell transcriptomic data.

To further increase immune cell identification power, we introduced an additional metric of total immune gene expression, defined as the sum of each cell’s LM22 gene expressions. As expected, immune cells’ total immune gene expressions were much greater than non-immune cells (Fig. 1d). The immune and non-immune cells were clearly divided in the plane of the maximum correlation coefficient and total immune gene expression (Fig. 1e). Using a line as the boundary (dotted line in Fig. 1e), we were able to achieve 97.7% [97.67–97.71%] of sensitivity and 98.3% [98.23–98.30%] of specificity (see the Receiver Operating Characteristic (ROC) curve in Fig. 1f).

ImmunIC’s identification power surpassed the conventional clustering method^7^. While immune cells were separated from non-immune cells in the reduced dimensional space by clustering, around 75% of pancreatic cells from one study^55^ clustered with myeloid cells and PBMCs (Fig. 1g). Around 32% of brain tumor cells and 27% of breast cancer cells were also grouped with immune cells. Additionally, plasma cells formed a separate cluster from other immune cells (dotted circle in Fig. 1g). These limitations clearly suggest that the conventional clustering approach is not desirable for the identification of immune cells from multicellular specimens including non-immune cells. Usage of the leukocyte gene signature matrix^14^ provided systematic identification of immune cells by capturing transcriptomic signatures of immune cells that are conserved across different datasets.

### Immune cell type annotation by ImmunIC

**Figure 2:**
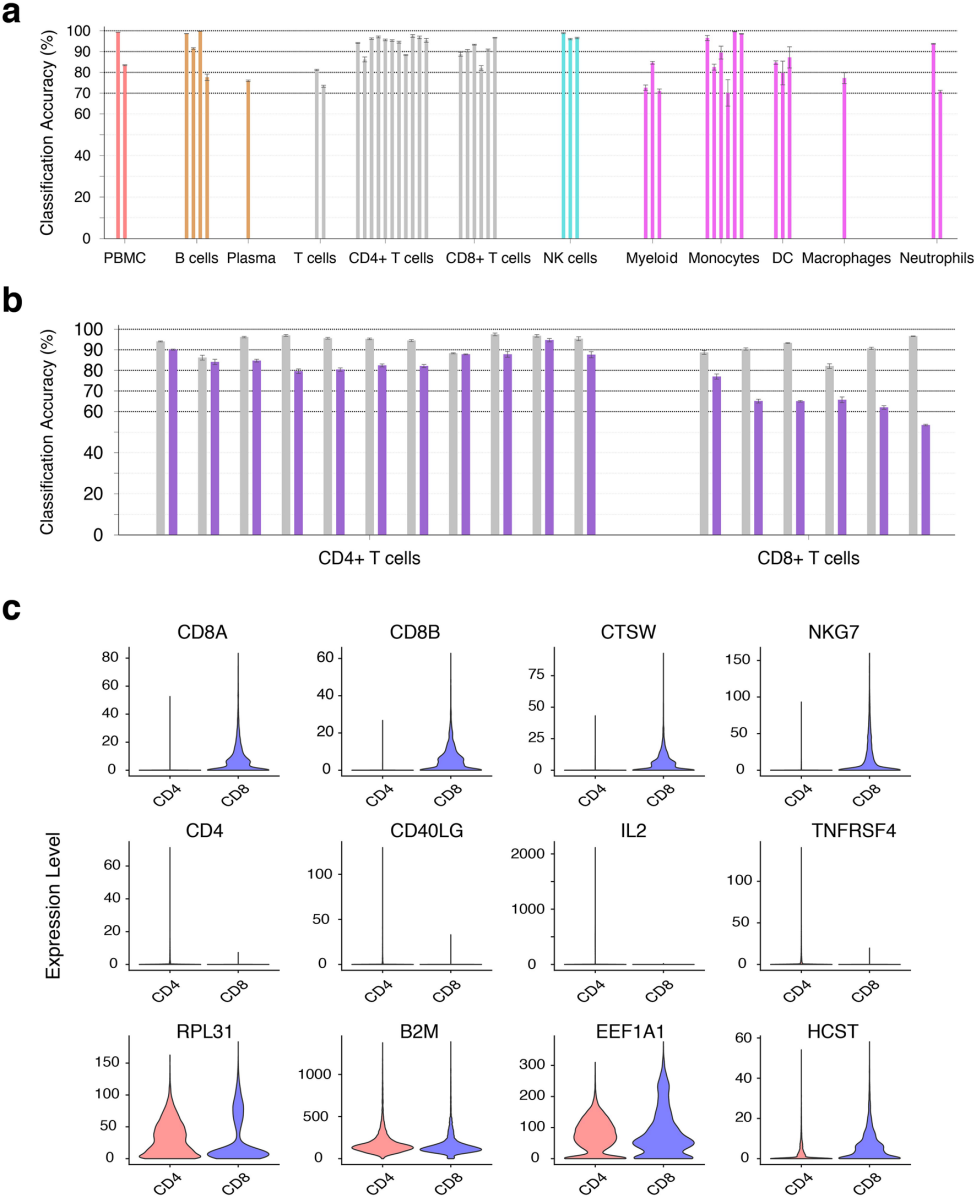
ImmunIC’s immune cell classification accuracy. (**a**) The accuracy of ImmunIC, which is the proportion of immune cells that are correctly classified as their respective cell types, is plotted for each single cell RNA sequencing dataset. Each bar corresponds to the accuracy of a specific dataset including PBMC (PBMC-1 and PBMC-2), B cells (from BC-1 to BC-4), Plasma (PLASMA), T cells (TC-1 and TC-2), CD4+ T cells (from CD4-1 to CD4-11), CD8+ T cells (from CD8-1 to CD8-6), NK cells (from NK-1 to NK-3), Myeloid (from Myeloid-1 to Myeloid-3), Monocytes (from Mono-1 to Mono-6), DC (from DC-1 to DC-3), Macrophages (Macro), and Neutrophils (Neutro-1 and Neutro-2), as ordered in Table 1. The 95% confidence interval is provided alongside each bar. (**b**) CD4+ and CD8+ T cell classification accuracy with (grey) and without (purple) the Xgboost classifier. (**c**) Important features identified by the Xgboost classifier include conventional CD8+ T cell markers (top row), CD4+ T cell markers (middle row) and non-conventional genes (bottom row).

ImmunIC assigns an immune cell as either B cell, plasma cell, T cell, NK cell, monocyte, dendritic cell, macrophage, neutrophil, or other myeloid cell group based on the maximum correlation coefficient to LM22. When a cell is assigned to T cell, ImmunIC further classifies into either CD4+ or CD8+ T cell by an Xgboost classifier^16^. Our simple and automated workflow showed 91.6% [91.6–91.7%] of immune cell-type classification accuracy for around one million cells. As presented in Fig. 2a and Table 1, the classification accuracy ranged from 70.1% to 99.8% for 42 datasets collected from 29 different studies. Over 300,000 B cells from two independent studies (BC-1 and BC-3) were labeled as B cells with around 99% accuracy. However, the accuracy for plasma cells was lower since around 20% of these were annotated as non-immune cells. The classification accuracy of CD4+ and CD8+ T cells was in the range of 82% and 98%. Notably, around 30,000 NK cells showed more than 96% accuracy across three independent studies. On contrary, around 8% of 4,434 myeloid cells (Myeloid-1) were misclassified as non-immune cells and 11% and 8% of those were misclassified as B cells and T cells, respectively. The confusion matrix of ImmunIC was presented in Supplementary Table S1. Although there is variation in the classification accuracy, the overall accuracy of ImmunIC was over 90% across one million single cells.

The addition of an Xgboost classifier enhanced CD4+ and CD8+ T cell classification accuracy from 78 to 93%, on average (p < 0.001, Fig. 2b). To address gene expression profile heterogeneity across different studies, we trained a classifier with 10,000 CD4+ T cells that were randomly selected from ten different studies (from CD4-1 to CD4-10 in Table 1) and 5,000 CD8+ T cells from five studies (from CD8-1 to CD8-5). We further examined the Xgboost classifier’s accuracy by five-fold cross-validation. The average classification accuracy over five independent tests was highly desirable, ranging from 93.6% to 99.9% (Supplementary Table S2). We also tested two additional datasets, 1,886 CD4+ T cells (CD4-11) and 69,457 CD8+ T cells (CD8-6), which were not included in any of our training sets. Our trained models showed 99.8% accuracy for CD4-11 and 99.7% accuracy for CD8-6.

A total of 334 genes were identified as important features that include CD4+ T cell markers (CD4, CD40LG, IL2 and TNFRSF4) and CD8+ T cell markers (CD8A, CD8B, CTSW, and NKG7), as presented in Fig. 2c. Important features also included genes that have not been linked to CD4+ and CD8+ T cell markers (Fig. 2c). We found that the designated genes were associated with differential signaling pathways of CD4+ and CD8+ T cells, including Th1 and Th2 Activation pathway with 9.3% coverage of DEGs (CD4, CD40LG, CD86, CD8A, GRB2, HLA-B, HLA-DRB5, IKZF1, IL2, IL2RA, IL2RG, JUN, KLRD1, S1PR1, TGFB1, and TNFRSF4), IL-2 Signaling pathway with 11.5% coverage (FOS, GRB2, IL2,I L2RA, IL2RG, JUN, and NRAS), and Granzyme A Signaling pathway with 10.5% coverage (H1-3 and H1-4).

### Comparison of ImmunIC with other immune cell classifiers

**Figure 3:**
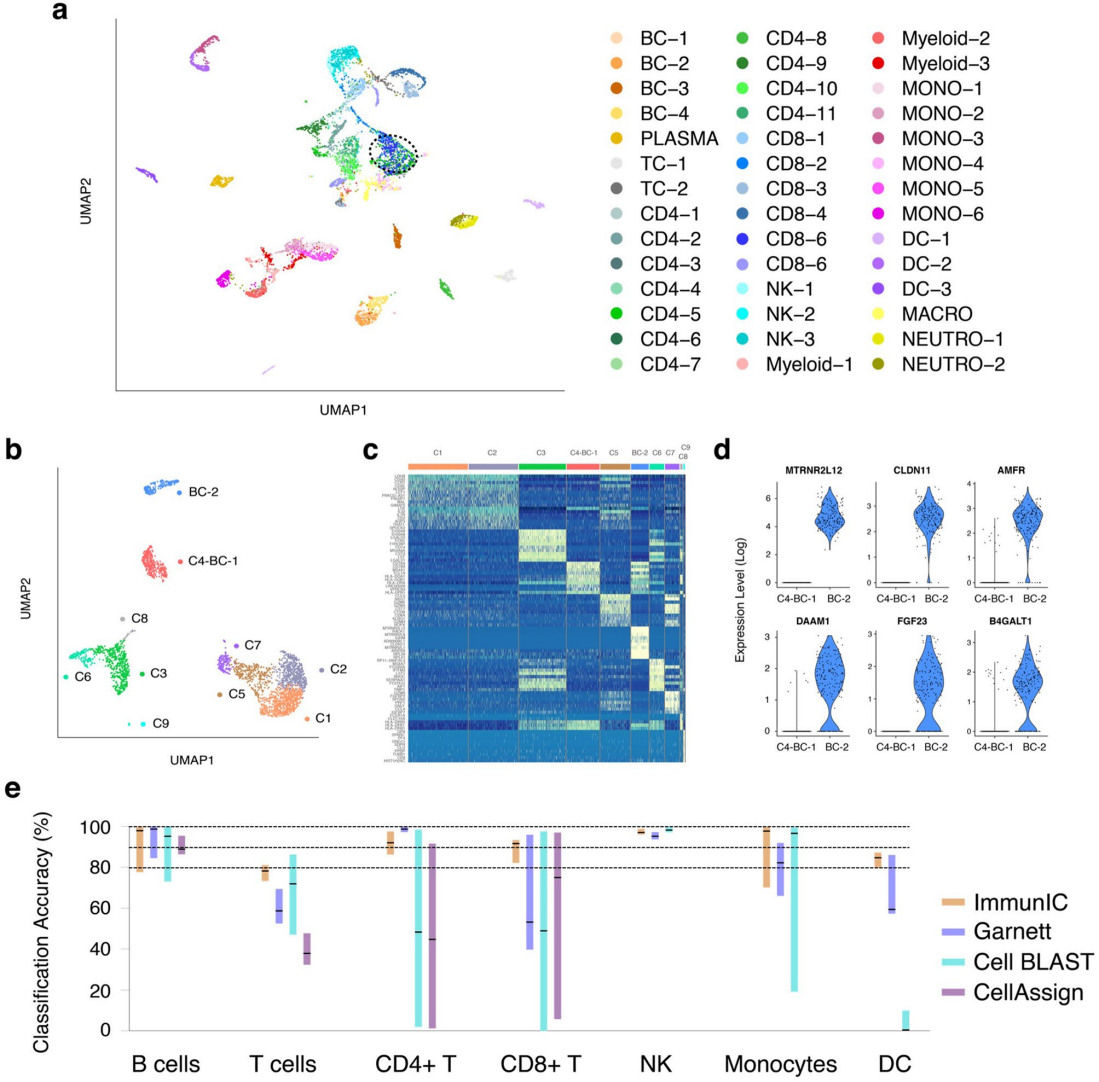
Limitations of clustering-based immune cell classification. (**a**) UMAP presentation of immune cells in each group of B cells (from BC-1 to BC-4), plasma cells (PLASMA), T cells (TC-1 and TC-2), CD4+ T cells (from CD4-1 to CD4-11), CD8+ T cells (from CD8-1 to CD8-6), NK cells (from NK-1 to NK-3), myeloid cells (from Myeloid-1 to Myeloid-3), monocytes (from MONO-1 to MONO-6), dendritic cells (from DC-1 to DC-3), macrophages (MACRO), neutrophils (NEUTRO-1 and NEUTRO-2). A total of 200 cells were randomly selected from each dataset (192 cells from DC-2 and 164 cells from DC-3) and these cells’ gene counts were combined for clustering analysis^56^ using Seurat 4.0^7^. Both CD4+ and CD8+ T cells were mixed together within the dotted circle. (**b**) UMAP presentation of 2,700 PBMCs from one individual^56^ along with 200 B cells from other study^5^. Added B cells formed a separate cluster (BC-2, colored by blue) from the PBMCs’ B cell cluster (C4-BC-1, colored by red). (**c**) Heatmap of DEGs of 2,700 PBMCs’ 9 clusters (from C1 to C9) with the added B cell cluster (BC-2). (**d**) Violin plots of upregulated genes among the added B cells (BC-2), compared to B cells within the PBMCs. (**e**) Classification accuracy for ImmunIC, Garnett, Cell BLAST, and CellAssign for seven groups of cells in Table 1. Each bar represents the minimum and maximum accuracy across each cell group’s studies. The average accuracy is marked by black line.

We directly compared ImmunIC’s performance with the conventional clustering method^7^. We conducted unsupervised clustering on the 42 immune cell datasets collected from 29 different studies (from BC-1 to Neutro-2 in Table 1). We observed that CD4+ and CD8+ T cells were mixed together in the reduced dimension. As shown in Fig. 3a, four groups of CD4+ T cells (CD4-3, CD4-5, CD4-6, and CD5-7 in Table 1) and two groups of CD8+ T cells (CD8-2 and CD8-5 in Table 1) were clustered together. Note that these six groups of cells are from a single study^17^ and these cells were grouped together by this study’s gene markers, rather than by CD4+ and CD8+ T cell differential markers. Our observation was consistent with a recent study reporting that CD4+ and CD8+ T cells were not clearly separated at a single-cell transcriptome level^7^. Similarly, one group of dendritic cells (DC-2) was clustered with monocytes (MONO-3) from the same study^43^, not with dendritic cells from other studies (Fig. 3a). Taken together, study-specific gene markers are important factors for determining clustering outcomes and we were not able to obtain cell-type specific clusters with the conventional clustering method.

Study-specific gene expression signatures dictated clustering outcomes even when only two datasets were analyzed together. Clustering of 2,700 PBMCs from an individual previously identified 9 immune cell clusters including B cells, CD8+ T cells and NK cells^56^. When we added B cells from another study^5^, these B cells did not belong to the PBMCs’ B cell cluster (C4-BC-1 in Fig. 3b) but rather formed a separate cluster (BC-2 in Fig. 3b). This was because the added B cells had markers that defined a separate cluster, as shown in a heatmap of differentially expressed genes (DEGs) among clusters (Fig. 3c). The added B cells showed high expressions for B cell markers, CD79A and CD79B. However, other genes such as RACK1 and RPL39 were uniquely expressed, creating a separate cluster (Fig. 3c). Figure 3d compared expressions of six genes between B cells of the PBMC cluster and the added B cells. Likewise, NK cells from other study^38^ did not cluster with the PBMCs’ NK cells (Supplementary Fig. S3). This clustering-based two-dimensional representation failed to group together the same type of immune cells from different studies, limiting its annotation capacity.

We next compared ImmunIC’s performance with three other immune cell classifiers: Garnett^8^, Cell BLAST^10^, and CellAssign^9^. Figure 3e presented each method’s minimum and maximum classification accuracy across different studies in each of seven cell groups (see Table 1 and Supplementary Table S3). ImmunIC outperformed other methods for both T cell and CD8+ T cell assignments (Fig. 3e). ImmunIC showed 92% classification accuracy for CD8+ T cells on average while the accuracy of Garnett (53%), Cell BLAST (49%), and CellAssign (75%) was significantly lower for the same cell population. Notably, the other methods showed a large variability in accuracy across different single cell RNA sequencing datasets. The minimum classification accuracy for CD8+ T cells was 39% for Garnett, 0% for Cell BLAST and 6% for CellAssign. These values were considerably lower than ImmunIC’s minimum accuracy of 82%. ImmunIC also performed better in designating monocytes and dendritic cells than Garnett and Cell BLAST (Fig. 3e and Supplementary Table S3). ImmunIC’s accuracy for six groups of monocytes ranged from 70 to 100%, which was higher than that of Garnett (from 66 to 92%) and Cell BLAST (from 19 to 99%). The minimum accuracy for three groups of dendritic cells was 85% for ImmunIC, 57% for Garnett and 0.1% for Cell BLAST. Note that CellAssign does not designate monocytes and dendritic cells as a separate population. Overall, ImmunIC showed the best performance in immune cell classification with its average accuracy of 93%, compared to the existing algorithms of Garnett (84%), Cell BLAST (74%), and CellAssign (69%).

### PBMC classification by ImmunIC

**Figure 4:**
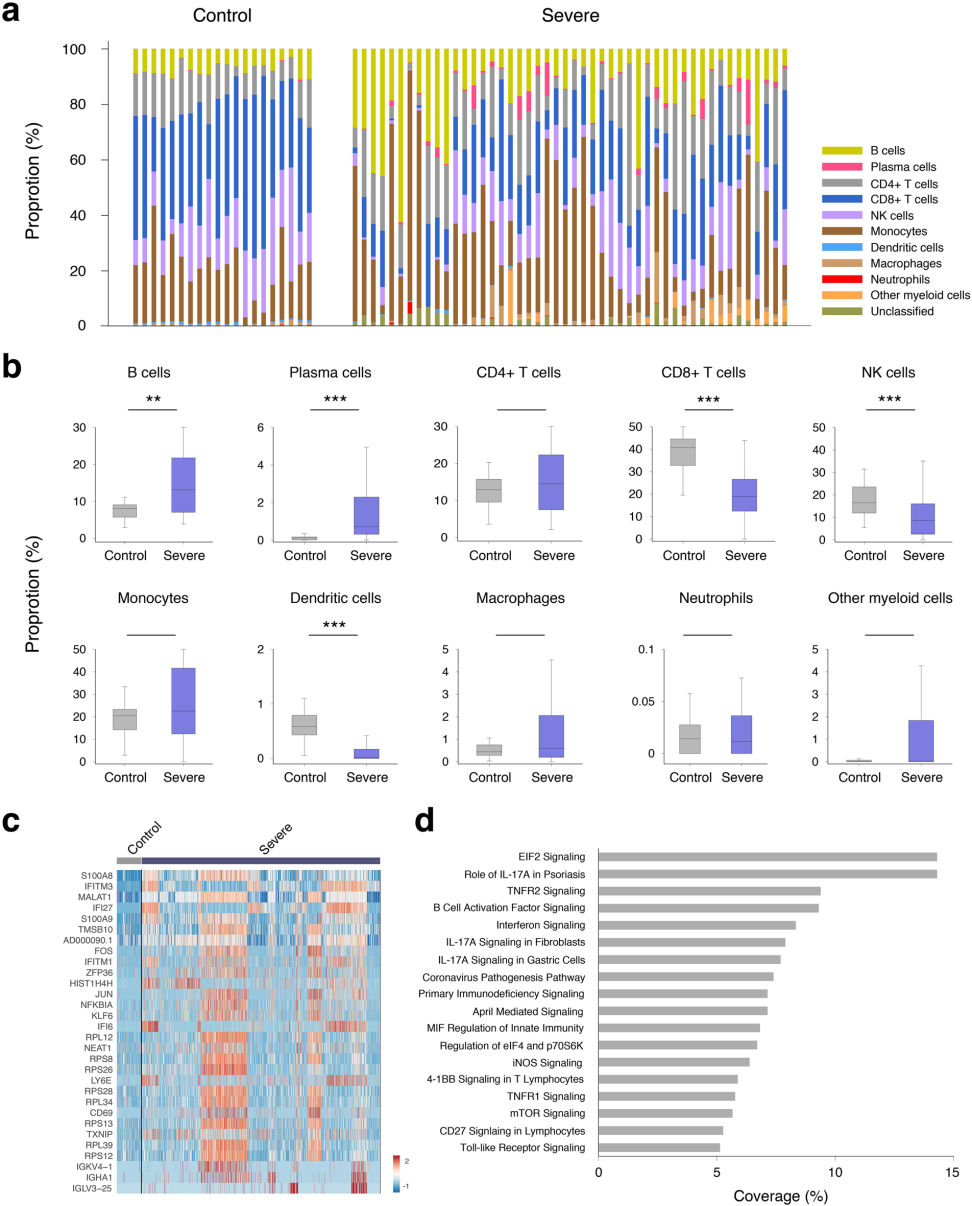
PBMC compositions determined by ImmunIC. (**a**) PBMC composition profiles of 48 individuals at COVID-19 severe progression and 20 healthy controls, determined by ImmunIC. (**b**) Box plots of each cell type proportion between the severe and healthy control groups. Proportions of B cells and plasma cells were significantly higher in the severe progression group than the control (p = 0.0015 and p < 0.001). Proportions of CD8+ T cells, NK cells, and dendritic cells were lower in the severe progression group than the control (p < 0.001, p < 0.001, and p < 0.001). (**c**) Heatmap of top 30 upregulated genes in 6,001 macrophages in the severe progression group (ordered by adjusted p value), compared to 615 macrophages in the control group. (**d**) Pathways that are upregulated in the severe group’s macrophages compared to the control group, with greater than 5% coverage of DEGs.

ImmunIC analyzed previously published single cell RNA sequencing data of 48 individuals who were at the severe progression state of COVID-19 infection and 20 healthy controls^5^ and determined the PBMC composition of these individuals. Figure 4a shows the PBMC profiles measured from around 6,000 PBMCs from each of 68 individuals. As in Fig. 4b, there was a greater than tenfold increase of plasma cells in the severe group (0.13% vs. 2.2%, p < 0.001), as reported in the original publication^5^. While the proportion of B cells was elevated in severe patients (7.5% vs. 17.4%, p = 0.0015), the percentage of lymphocytes was significantly decreased (78.6% vs. 65.8%, p = 0.039). The observed decrease is in line with lymphopenia, a hallmark of severe COVID-19^57^. In particular, both CD8+ T cells and NK cells were significantly reduced within the PBMC population of severe COVID-19 patients (40.3% vs. 19.5%, p < 0.001 and 18.1% vs. 10.6%, p < 0.001). The percentage of dendritic cells was also lower in the severe group than the control (0.6% vs. 0.16%, p < 0.001). Severe COVID-19 cases’ PBMC profiles determined by ImmunIC agreed with polychromatic flow cytometry-based immunophenotyping measures^58^.

We then conducted functional pathway analysis on each immune cell population using the Ingenuity Pathway Analysis (IPA) program. Figure 4c shows the top 30 upregulated DEGs of macrophages in the severe group versus the control. Inflammatory genes including IFI27, FOS, JUN and NKFBIA were significantly upregulated in the severe group. Signaling pathway analysis highlighted that pro-inflammatory pathways are upregulated in the macrophages of the severe group, including IF-17A pathways (S100A8, S100A9, FOS, JUN, NKFBIA), TNFR2 Signaling pathway (FOS, JUN, NFKBIA), and Interferon Signaling pathway (IFI6, IFITM1, and IFITM3). Figure 4d plotted the severe group’s differential pathways with greater than 5% coverage of macrophages’ DEGs (see Supplementary Table S4 for more details). In addition, EIF2 Signaling pathway was enriched in severe cases with 14.3% coverage of differential genes such as RPL10, RPL11, and RPL12.

Monocytes identified by ImmunIC showed both similar and dissimilar transcriptomic signatures of COVID-19 severity compared to macrophages. Role of Hypercytokinemia/hyperchemokinemia in the Pathogenesis of Influenza pathway was found to be significantly upregulated in the severe group’s monocytes with DEGs of AREG, CCL3, CCL4, CXCL8, IL1B, and ISG15 (coverage = 7% and p < 0.001). In addition, Airway Inflammation in Asthma pathway (CXCL8 and RNASE2) was enhanced in the monocyte population. Similar to macrophages, monocytes in the severe group showed enhanced Interferon Signaling pathway with DEGs of IFI6, IFITM1, IFITM3, and ISG15 (coverage = 11%, p < 0.001). Supplementary Table S5 lists monocyte-specific differential pathways of COVID-19 severe cases.

## Discussion

ImmunIC (Immune cell Transcriptome Classifier) is a simple and automated tool for immune cell identification and classification from a mixture of immune and non-immune cells. ImmunIC showed significant consistency in immune cell identification and classification accuracy across 66 independent single cell RNA sequencing studies. ImmunIC’s two metrics, maximum correlation coefficient and total immune gene expression, provided a high immune cell identification accuracy of 98%. We then used the maximum correlation coefficient to assign immune cell type and further increased the accuracy using an Xgboost classifier. The accuracy of ImmunIC’s immune cell type classification was around 92% for over one million immune cells.

ImmunIC outperformed existing immune cell classifiers^7–10^. ImmunIC was robust in assigning a massive number of diverse immune cells with the minimum of 70% accuracy. When the existing methods were benchmarked with the same datasets, we observed a large variability in accuracy, marking the minimum accuracy of 40% (Garnett^8^), 0% (Cell BLAST^10^), and 1% (CellAssign^9^). In order to properly handle highly variable study-specific single cell transcriptomic signatures, we trained our classification model with datasets collected from multiple studies. Therefore, our model can be readily used without requiring further trainings. Indeed, ImmunIC showed a high level of classification accuracy for datasets which were not included in our training.

Our classifier clearly showed a capacity to sort immune cells directly from single cell transcriptomic readouts. Healthy individuals’ PBMC compositions determined by our immune cell classifier agreed with previous phenotypic measurements. We estimated the frequency of lymphocytes (B cells, T cells and NK cells) as 78.6%, which is consistent with that measured by polychromatic flow cytometry^59^. Among lymphocytes, B cells, T cells and NK cells were estimated to be 9.9%, 67.2% and 23%, respectively. The average frequency of monocytes (19.9%) was also consistent with previous reports^59^. The proportion of dendritic cells was estimated to range from 0% to 1.1% among 20 healthy individuals, which overlaps with the immunophenotypic measure of 1–2%^59^.

We demonstrated the utility of ImmunIC by performing signaling pathway analysis of a specific immune cell population. We identified functional pathways in macrophages that are upregulated among patients with severe COVID-19, compared to those of healthy controls. Most of the identified pathways, including TNFR2 Signaling, B cell Activating Factor Signaling, and IL-17A Signaling, showed upregulation of three key inflammatory genes: JUN, FOS, and NFKB1A. These pathways are critical to the activation of macrophages and induction of inflammatory responses to COVID-19 infection^60,61^. In addition, Interferon Signaling pathway was enhanced in the macrophage population, as previously reported^61^. We also observed that EIF2 Signaling (associated with mRNA translation) is significantly upregulated in the macrophages of the severe group. Upregulation of EIF2 Signaling was previously reported in severe patients’ lung specimens where macrophage infiltration was confirmed^62^. We found that Role of Hypercytokinemia/hyperchemokinemia in the Pathogenesis of Influenza pathway was enhanced in severe cases’ monocytes, corroborating a study of the monocyte-driven cytokine storm in SARS-CoV-2 infections^63^. Taken together, our high-resolution classifier allows immune cell-type specific functional pathway analysis.

ImmunIC achieved accurate sorting of immune cells into ten distinct categories, including a clear separation between CD4+ and CD8+ T cells. However, further annotation into more specific immune cell types poses challenges. For instance, only 60% of memory B cells (BC-2) were assigned as memory B cells by the maximum correlation to LM22, while ImmunIC labeled 92% of these cells as B cells. Similarly, only three percent of regulatory CD4+ T cells (CD4-5) were correctly classified as regulatory cells. Therefore, we limited the number of cell types as 10 based on LM22’s 22 immune cell type profiles. As more data becomes available, the Xgboost classifier can be expanded to annotate subcategories of each immune cell. Alternatively, novel marker genes could be proposed to enhance the resolution for identifying more diverse immune cell types.

Our immune cell classification method is a time-efficient and accurate approach to designate ten immune cell populations from multicellular blood and tissue specimens. ImmunIC takes a single input of a gene count matrix, obtained from diverse single cell RNA sequencing platforms, to identify immune cells and determine immune cell composition by a single command line. In this way, ImmunIC is highly standardized and reproducible, not requiring any batch-specific modification on input data. ImmunIC can therefore serve as a reliable cell sorter for cell-type specific immune profiling.

## Methods

### Immune cells and non-immune cells’ single cell RNA sequencing data

Human single cell RNA sequencing data of 2,078,671 immune and 509,300 non-immune cells were collected from 66 previous publications. B cells analyzed in this study include a group of memory B cells (BC-2). CD4+ T cells include CD4+ Naïve T cells (CD4-6), CD4+ Memory T cells (CD4-7 and CD4-8) and regulatory CD4+ T cells (CD4-5, CD4-9 and CD4-10). The dataset also includes sorted CD8+ Naïve T cells (CD8-5). Monocytes include both classical and non-classical monocytes. Table 1 summarizes each study’s accession number, number of cells, and ImmunIC’s classification accuracy for immune cells. We analyzed 22 different cell types of non-immune cells: intestine cells, kidney cells, airway epithelial cells, prostate cells, oligodendrocytes, pancreatic islets cells, keratinocytes, neuroblastoma cells, retina cells, melanoma cells, entorhinal cortex cells, liver progenitor-like cells, mesothelial cells, breast epithelial cells, neuroendocrine tumor cells, oocytes, fibroblasts, oligodendroglioma cells, medulloblastoma cells, brain tumor cells, breast cancer cells and substantia nigra cells (Table 2).

### Maximum correlation coefficient and total immune gene expression of ImmunIC

Each study’s raw gene count matrix from single cell RNA sequencing data was used as input. Cells were filtered out when the total count was less than 500. Subsequently, each cell’s total count was normalized to 10,000. We first measured Pearson’s correlation coefficients of each cell’s gene expression (normalized count) to LM22’s 22 cell-type gene expression profiles. The maximum correlation coefficient is the maximum of these 22 values. For instance, 49 genes including ABCB9, MANEA, and MZB1 are marked as 1 and other 498 genes are marked as 0 in LM22’s plasma cell profile^14^. The total immune gene expression of each cell is defined as the sum of each cell’s 547 gene expressions of LM22. Here the total immune gene expression denotes the relative proportion of LM22’s immune gene expression since each cell’s total count is normalized to 10,000.

The maximum correlation coefficient and total immune gene expression were used as two metrics for immune cell identification. An input cell was designated as an immune cell when a linear sum of the maximum correlation coefficient (${\rho }_{\mathrm{max}})$ and total immune gene expression ($T$) is greater than a threshold ($\theta )$,

1 $$A\times {\rho }_{\mathrm{max}}+T> \theta .$$

By maximizing the sensitivity (proportion of immune cells that are classified as immune cells) and specificity (proportion of non-immune cells that are classified as non-immune cells) of 2,078,671 immune and 509,300 non-immune cells, $A$ was determined as 1580.7 and $\theta$ as 413 with the area under the ROC curve of 0.987.

When an input cell is classified as an immune cell by Eq. (1), it is further categorized into B cell, plasma cell, T cell, NK cell, monocyte, macrophage, dendritic cell, neutrophil and other myeloid cell based on the maximum correlation coefficient to LM22 profiles. When the ainput cell is designated as a T cell, it is inputted into an Xgboost classifier to further classify it as either CD4+ or CD8+ T cell. ImmunIC’s entire process is integrated as a single command line with a single input of gene-cell count matrix. ImmunIC demonstrates a rapid cell annotation capacity while requiring minimal computational power. It took only 4 min 56 s to annotate 5,300 cells (CD4-4), and 12 min 3 s to annotate 10,047 cells (BC-3) using a single thread on an Intel Xeon CPU E5-2620.

### Xgboost classifier of ImmunIC

One thousand cells were randomly selected from each of 10 different CD4+ T cell datasets (from CD4-1 to CD4-10 in Table 1) and 5 different CD8+ T cell datasets (from CD8-1 to CD8-5 in Table 1). The combined count matrix of 15,000 cells was log-normalized and used as an input for an Xgboost classifier. The Xgboost python module, *xgbclassifier.fit,* was used to fit a gradient boosting classifier with *max_depth* = 3, *eta* = 0.01, *lambda* = 0, *gamma* = 0.1, *alpha* = 0.5, *nthread* = 16, *subsample* = 0.5, and *importance_type* = gain. The trained model was then saved using *pickle.dump*. Important features were obtained using the module, *xgbclassifier.feature_importances_*. A total of 334 genes with a feature importance score greater than 10^–7^ were used for the classification step. Lastly, the module *predict* was used to return the classification outcome of either CD4+ or CD8+ T cell.

### Five-fold cross validation test

Five-fold cross-validation of the Xgboost classifier was conducted by dividing CD4+ and CD8+ T cells into training and test sets. A total of 1000 cells were randomly sampled from each of ten CD4+ T cell datasets (from CD4-1 to CD4-10) and these 10,000 cells were combined with 5,000 cells randomly picked from five CD8+ T cell datasets (from CD8-1 to CD8-5). The model was then trained with these 15,000 cells and tested by cells that were not included in the training set. This process was repeated five times such that each training data set was randomly sampled while the remaining cells were used as the test data. Two additional datasets, CD4-11 and CD8-6, were not included in any of our training procedures but tested by five independently trained models.

### Other immune cell classification algorithms

Using the R library *garnett*^8^, each cell’s size factor was estimated with the function, *estimateSizeFactors*. Cells were then assigned by *cluster_ext_type* from the function, *classify_cells*, using Garnett’s pre-trained classifier for PBMC, *hsPBMC_20191017.RDS* (downloaded from https://cole-trapnell-lab.github.io/garnett/classifiers/).

The python package, *Cell_BLAST*^10^, was used along with the reference panel, *Zheng.h5* (downloaded from https://cblast.gao-lab.org/download). Each study’s raw gene count matrix was loaded by *cb.data.ExprDataSet.read_table* and cells were annotated by *cell_ontology_class*.

The R library, *cellassign*^9^ was used with an input of cell-by-gene matrix of raw counts. Each cell’s size factor was estimated by *sizeFactors* in the R library, *SingleCellExperiment*. CellAssign’s marker gene set, *example_TME_markers*, was used to assign cell types using the function, *cellassign*.

### Signaling pathway analysis

A total of 6,001 cells were labeled as macrophages by ImmunIC from 276,605 PBMCs of 48 individuals at COVID-19 severe progression state while a total of 615 macrophages were identified from 126,799 PBMCs of 20 uninfected individuals. With the *FindAllMarkers* module of Seurat 4.0^7^, a total of 82 genes were obtained as macrophages’ upregulated DEGs in the severe progression group, compared to the control (log-fold-change > 1 and p_adj_ < 0.001). These genes were used as input for Ingenuity Pathway Analysis (IPA, Qiagen). Identified differential pathways with greater than 5% coverage of DEGs were listed in Supplementary Table S4.

A total of 74,645 cells were annotated as monocytes by ImmunIC from the 48 severe cases and a total of 24,152 monocytes were identified from the 20 healthy individuals. These two groups’ monocytes were compared using the *FindAllMarkers* module of Seurat 4.0^7^ and a total of 26 genes were found to be upregulated in the severe progression group compared to the control (log-fold-change > 1 and p_adj_ < 0.001). Monocytes’ differential pathways were then obtained by IPA, as listed in Supplementary Table S5.

## Supplementary Information

Supplementary Information.

## Data Availability

All single cell RNA sequencing datasets analyzed in the current study were downloaded from public repositories. Accession numbers along with source publications are provided in Tables 1 and 2.

## Code availability

ImmunIC is available at https://github.com/hayounlee-lab/immunic.
