# Supplementary material for: Immune cell identifier and classifier (ImmunIC) for single cell transcriptomic readouts
Source: Sci Rep. 2023 Jul 26;13:12093. doi: 10.1038/s41598-023-39282-4 (PMC10372073; doi:10.1038/s41598-023-39282-4)
Supplement: Supplementary file 1 — Supplementary Information. [file 41598_2023_39282_MOESM1_ESM.pdf]

Supplementary Information

Immune cell identifier and classifier (ImmunIC) for single cell transcriptomic readouts

Authors:

Sung Yong Park[1], Sonia Ter-Saakyan[1], Gina Faraci[1], and Ha Youn Lee[1*]

Affiliations:

[1]Department of Molecular Microbiology and Immunology, Keck School of Medicine, University of Southern California, Los Angeles, USA

Supplementary Figures

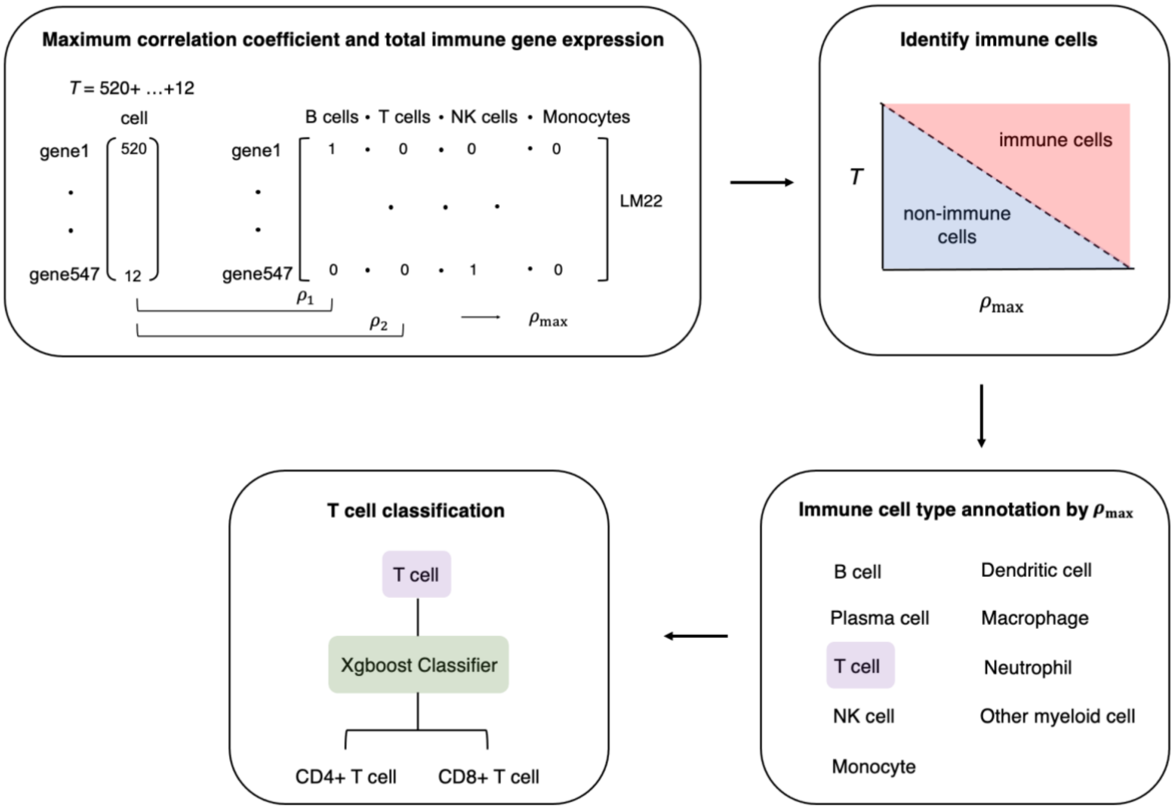

Supplementary Fig. S1 ImmunIC's workflow. The total immune gene expression (*T*) of an input cell is determined by summing the expression levels of 547 genes from its normalized single-cell RNA sequencing data, using the predetermined leukocyte gene signature matrix[1] (LM22). To calculate the correlation coefficient, each cell's normalized gene expression is compared to the gene profiles of various immune cell types in LM22, and the maximum correlation coefficient ($\rho_{\max}$) across different cell types is obtained. Immune cells were then differentiated from non-immune cells in the $\rho_{\max}$ and *T* plane, separated by the dotted line. If the input cell is classified as an immune cell, it is further categorized into B cell, plasma cell, T cell, NK cell, monocyte, macrophage, dendritic cell, neutrophil and other myeloid cell based on the maximum correlation coefficient to LM22 profiles. If the input cell is designated as a T cell, it is inputted into an Xgboost classifier to further classify it as either CD4+ or CD8+ T cell.

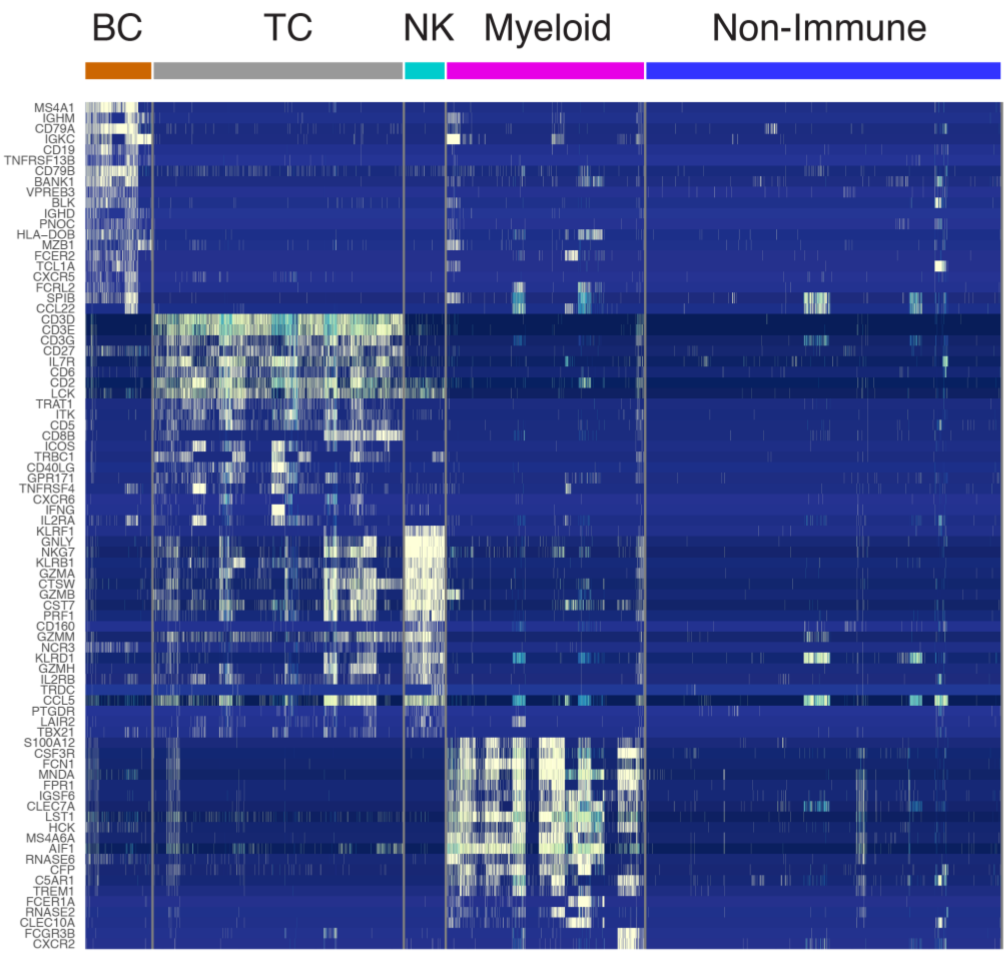

Supplementary Fig. S2 Differential gene expressions of immune and non-immune cells. Top 20 upregulated genes from LM22 547 genes for B cells, T cells, NK cells and myeloid cells (ordered by adjusted p value).

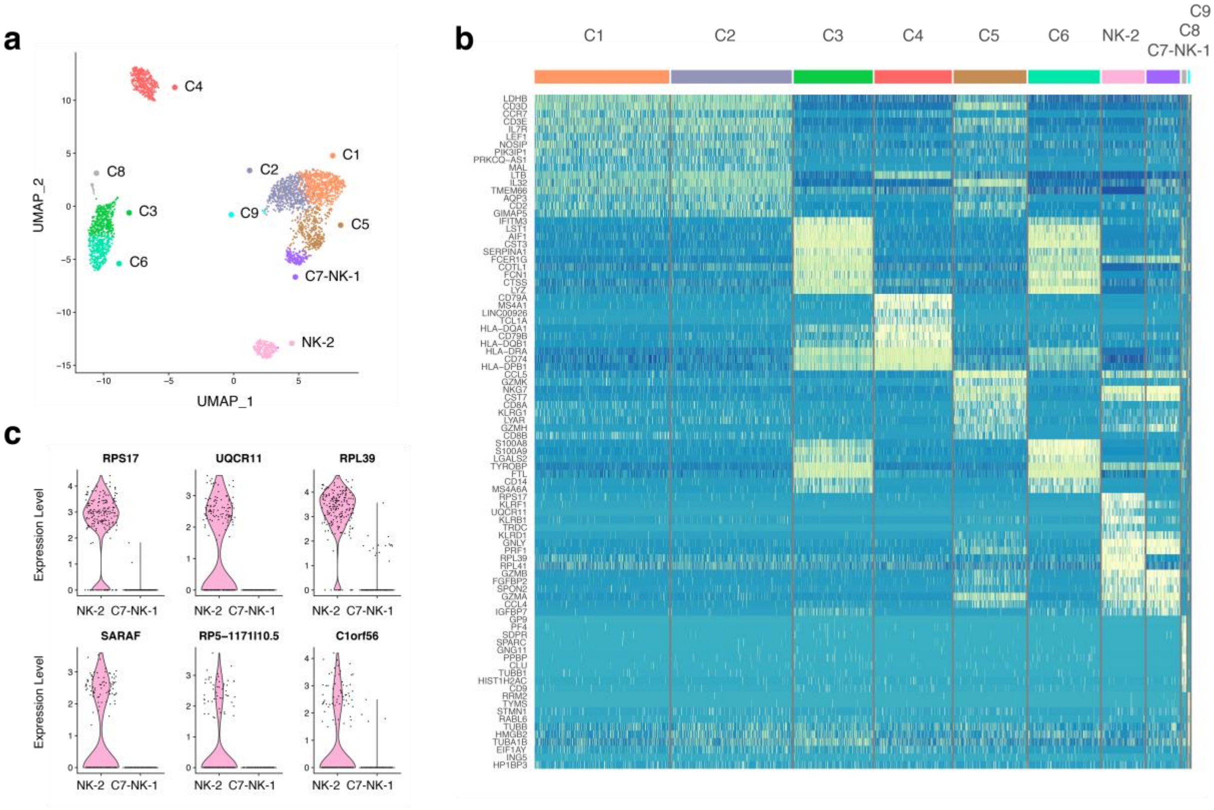

Supplementary Fig. S3. **Clustering of PBMCs and NK cells. a** UMAP presentation of 2,700 PBMCs from one individual[2] along with 200 NK cells from other study[3]. Added NK cells formed a separate cluster (NK-2, colored by pink), not grouping with PBMC's NK cells (C7-NK-1, colored by purple). **b** Heatmap of 9 clusters of 2,700 PBMCs (from C1 to C9) along with the added NK cells (NK-2). **c** Upregulated genes of the added NK cells (NK-2), compared to NK cells within the PBMCs (C7-NK-1).

Supplementary Tables

Study ID	B	Plasma	CD4+ T	CD8+ T	NK	Monocyte	DC	Macrophage	Neutrophil	Other myeloid	Non-immune cells
BC-1	97.3%	1.31%	0.11%	0.3%	0.24%	0.6%	0.015%	0.052%	0.001%	0.006%	0.083%
BC-2	91%	0.44%	0.3%	1.25%	0.08%	0.024%	0.26%	0.084%	0.02%	0.02%	6.5%
BC-3	99.8%	0.01%	0.03%	0%	0%	0%	0.01%	0%	0%	0%	0.16%
BC-4	75.9%	1.67%	1%	0.074%	0.15%	0%	1.7%	1.74%	0.037%	0.63%	17.1%
PLASMA	1.25%	75.9%	0.48%	0.56%	0.14%	0.52%	0.04%	0.087%	0.021%	0.049%	20.9%
TC-1	0.17%	0.028%	68.6%	12.5%	5.87%	0.031%	0.4%	0.89%	0.038%	2.43%	9.06%
TC-2	1.74%	0.047%	25.2%	48%	5.05%	18.8%	0.27%	0.46%	0.044%	0.014%	0.37%
CD4-1	0.016%	0.002%	94.1%	0.11%	2.57%	0%	0.53%	0.74%	0.0023%	0.081%	1.83%
CD4-2	0.032%	0%	86.2%	0%	0.35%	0%	2.22%	0.16%	0.6%	0.13%	10.3%
CD4-3	0.5%	0.009%	96.2%	0.71%	0.18%	0%	0.027%	0%	0%	0.009%	2.36%
CD4-4	0.075%	0%	97%	0.6%	1.94%	0.057%	0.075%	0.094%	0%	0.094%	0.075%
CD4-5	0.69%	0%	95.6%	0.25%	0.33%	0%	0.03%	0%	0%	0.0098%	3.07%
CD4-6	0.45%	0.048%	95.3%	0.96%	0.12%	0%	0.0096%	0%	0%	0%	3.08%
CD4-7	0.29%	0.0098%	94.5%	1.26%	0.31%	0%	0.0098%	0%	0%	0.02%	3.6%
CD4-8	0.0014%	0%	88.3%	0.08%	6.73%	0%	0.51%	0.078%	0.029%	3.64%	0.58%
CD4-9	0.29%	0%	97.5%	0.14%	1.29%	0.048%	0.048%	0.095%	0%	0.14%	0.43%
CD4-10	0.41%	0%	96.8%	0.035%	0.21%	0%	0%	0%	0%	0%	2.56%
CD4-11	0%	0%	95.4%	0.21%	0.21%	0.11%	2.7%	0.8%	0%	0.21	0.32%
CD8-1	0%	0%	1.73%	88.7%	9.44%	0%	0.023%	0.023%	0%	0.023%	0.045%
CD8-2	0.069%	0%	5.08%	90.4%	3.07%	0%	0%	0%	0%	0%	1.34%
CD8-3	0.021%	0.019%	0.37%	93.3%	4%	0%	0.012%	0.1%	0.07%	0.13%	1.96%
CD8-4	0%	0%	0.16%	82.1%	15.2%	0.067%	0%	0%	0%	0%	2.45%
CD8-5	0.084%	0.0084%	5.7%	90.7%	0.076%	0%	0%	0%	0%	0%	3.43%
CD8-6	0.0086%	0%	0.26%	96.6%	1.26%	0%	0%	0.0043%	0.058%	0.0043%	1.8%
NK-1	0%	0%	0.3%	0.71%	98.8%	0.012%	0.012%	0%	0%	0%	0.19%
NK-2	0.018%	0%	1.77%	1.12%	96%	0.027%	0.0091%	0%	0%	0%	1.01%
NK-3	0%	0%	2.35%	0.89%	96.5%	0.011%	0%	0%	0%	0.011%	0.21%
Myeloid-1	6.36%	4.26%	7.74%	0.47%	0.56%	20.7%	25.8%	0.79%	0%	25.3%	8.03%
Myeloid-2	1.25%	0.97%	0.35%	0.41%	1.17%	82.3%	1.97%	0.089%	0.19%	0.12%	11.2%
Myeloid-3	1.69%	0.29%	1.76%	0.062%	0.35%	42.5%	19.6%	8.41%	0.22%	0.25%	24.8%
Mono-1	0.35%	0%	0.7%	0.23%	0.23%	96.5%	0.35%	0%	0.35%	0%	1.29%
Mono-2	0.38%	0.57%	0.65%	0.077%	0.27%	82.5%	5.21%	1.34%	0.73%	0.11%	8.12%
Mono-3	0%	0%	0.54%	0.27%	2.69%	89.5%	1.08%	0.27%	1.88%	1.08%	2.69%
Mono-4	0%	0%	0%	0%	0%	70.1%	0%	2.54%	3.55%	3.05%	20.8%
Mono-5	0%	0%	0%	0%	0%	99.6%	0.13%	0.064%	0.037%	0%	0.16%
Mono-6	0.51%	0.0058%	0.0029%	0.0029%	0.0058%	98.5%	0.046%	0.22%	0.22%	0.17%	0.36%
DC-1	0%	0%	0.014%	0%	0.014%	0%	84.7%	13.9%	0%	0.027%	1.33%
DC-2	0%	0%	0%	0%	0.52%	18.2%	79.7%	0.52%	0%	0%	1.04%
DC-3	0.61%	0%	0%	0%	0%	0%	87.2%	0%	0%	0%	12.2%
Macro	0%	0%	0.2%	0%	0.1%	8.51%	10.5%	77.2%	0.2%	0.2%	3.1%
Neutro-1<br>Neutro-2	0.026%	0.0033%	0.023%	0.0049%	1.84%	4.06%	0.0016%	0.011%	93.7%	0.25%	0.078%

Supplementary Table S1. Confusion matrix of ImmunIC.

Study ID	Study Accession Number	Cross Validation Test Accuracy
CD4-1	EGAS00001003215[4]	99.9% [99.8% - 99.9%]
CD4-2	GSE121267[5]	99.9% [99.9% - 100%]
CD4-3	[6]	99.3% [99.2% - 99.4%]
CD4-4	GSE99254[7]	99.3% [99.2% - 99.4%]
CD4-5	[6]	99.7% [99.7% - 99.8%]
CD4-6	[6]	99.1% [98.9% - 99.3%]
CD4-7	[6]	98.6% [98.5% - 98.7%]
CD4-8	GSE150132[8]	99.8% [99.7% - 99.9%]
CD4-9	GSE99254[7]	99.5% [99.4% - 99.7%]
CD4-10	GSE119373[9]	99.9% [99.8% - 100%]
CD4-11	GSE122846[10]	99.8% [99.7% - 99.9%]
CD8-1	GSE99254[7]	97.7% [97.5% - 97.8%]
CD8-2	[6]	93.6% [93.3% - 94.5%]
CD8-3	GSE180268[11]	99.5% [99.4% - 99.6%]
CD8-4	GSE169503[12]	99.7% [99.7% - 99.5%]
CD8-5	[6]	93.0% [92.5% - 93.5%]
CD8-6	GSE159252[13]	99.7% [99.6% - 99.8%]

Supplementary Table S2. Five-fold cross-validation of our Xgboost classifier. Average classification accuracy with minimum and maximum accuracy out of five independent tests.

Study ID	Number of Cells	ImmunIC	Garnett[14]	Cell BLAST[15]	CellAssign[16]
BC-1	294,643	98.6% [98.6% - 98.6%]	98.8% [98.7% - 98.8%]	96.4% [96.3% - 96.4%]	88.3% [88.2% - 88.4%]
BC-2	24,946	91.5% [91.1% - 91.8%]	98.8% [98.7% - 99.0%]	82.5% [82.0% - 83.0%]	95.4% [95.1% - 95.7%]
BC-3	10,047	99.8% [99.7% - 99.9%]	100% [100% - 100%]	100% [99.9% - 100%]	90.7% [90.1% - 91.3%]
BC-4	2,700	77.6% [76.0% - 79.2%]	84.5% [83.1% - 85.8%]	73.0% [71.3% - 74.6%]	86.3% [85.0% - 87.6%]
TC-1	63,861	81.1% [80.8% - 81.4%]	52.5% [52.1% - 52.9%]	86.2% [85.9% - 86.4%]	32.3% [31.9% - 32.6%]
TC-2	36,550	73.3% [72.8% - 73.7%]	69.4% [68.9% - 69.8%]	47.0% [46.5% - 47.5%]	47.7% [47.2% - 48.2%]
CD4-1	43,112	94.1% [93.9% - 94.3%]	97.2% [97.0% - 97.4%]	77.1% [76.7% - 77.5%]	54.0% [53.5% - 54.4%]
CD4-2	3,149	86.2% [85.0% - 87.4%]	100% [100% - 100%]	35.5% [33.8% - 37.2%]	90.0% [88.9% - 91.0%]
CD4-3	11,180	96.2% [95.9% - 96.6%]	99.9% [99.8% - 100%]	86.9% [86.3% - 87.5%]	6.1% [5.6% - 6.5%]
CD4-4	5,300	97.0% [96.5% - 97.4%]	100% [100% - 100%]	96.2% [95.7% - 96.7%]	1.2% [0.9% - 1.4%]
CD4-5	10,162	95.6% [95.2% - 96.0%]	100% [100% - 100%]	89.0% [88.4% - 89.6%]	6.4% [5.9% - 6.9%]
CD4-6	10,427	95.3% [94.9% - 95.7%]	99.2% [99.0% - 99.3%]	89.8% [89.2% - 90.4%]	91.6% [91.0% - 92.1%]
CD4-7	10,224	94.5% [94.1% - 94.9%]	99.3% [99.1% - 99.4%]	97.8% [97.5% - 98.1%]	5.8% [5.3% - 6.3%]
CD4-8	73,566	88.3% [88.1% - 88.6%]	98.8% [98.7% - 98.9%]	1.9% [1.8% - 2.0%]	51.2% [50.8% - 51.5%]
CD4-9	2,098	97.5% [96.9% - 98.2%]	100% [100% - 100%]	98.5% [98.0% - 99.0%]	37.9% [35.8% - 40.0%]
CD4-10	2,892	96.8% [96.1% - 97.4%]	100% [100% - 100%]	65.1% [63.4% - 66.9%]	10.7% [9.6% - 11.8%]
CD4-11	1,886	95.4% [94.5% -96.4%]	97.2% [96.4% -97.9%]	55.6% [55.3% - 57.8%]	75.0% [73.1% - 77.0%]
CD8-1	4,439	88.7% [87.8% - 89.6%]	52.4% [50.9% - 53.9%]	0% [0% - 0%]	82.5% [81.3% - 83.6%]
CD8-2	10,192	90.4% [89.9% - 91.0%]	68.5% [67.6% - 69.4%]	96.5% [96.2% - 96.9%]	43.9% [42.9% - 44.9%]
CD8-3	56,470	93.3% [93.1% - 93.5%]	57.5% [57.1% - 57.9%]	22.4% [22.0% - 22.7%]	97.0% [96.9% - 97.1%]
CD8-4	4,487	82.1% [81.0% - 83.2%]	61.4% [59.9% - 62.8%]	51.4% [50.0% - 52.9%]	96.9% [96.4% - 97.4%]
CD8-5	11,915	90.7% [90.2% - 91.2%]	95.9% [95.6% - 96.3%]	97.6% [97.3% - 97.8%]	5.7% [5.3% - 6.1%]
CD8-6	69,457	96.6% [96.5% - 96.7%]	39.7% [39.3% - 40.1%]	58.1% [57.7% - 58.4%]	71.7% [71.3% - 72.0%]
NK-1	8,339	98.8% [98.5% - 99.0%]	95.0% [94.5% - 95.4%]	99.5% [99.3% - 99.6%]	NA
NK-2	11,036	96.0% [95.7% - 96.4%]	93.6% [93.2% - 94.1%]	97.1% [96.8% - 97.4%]	NA
NK-3	9,195	96.5% [96.2% - 96.9%]	97.2% [96.9% - 97.5%]	98.5% [98.3% - 98.7%]	NA
Mono-1	856	96.5% [95.3% - 97.7%]	89.7% [87.7% - 91.8%]	91.4% [89.5% - 93.2%]	NA
Mono-2	2,612	82.5% [81.1% - 84.0%]	87.0% [85.7% - 88.3%]	94.4% [93.5% - 95.3%]	NA
Mono-3	372	89.5% [86.4% - 92.6%]	67.2% [62.4% - 72.0%]	19.1% [15.1% - 23.1%]	NA
Mono-4	197	70.1% [63.7% - 76.4%]	66.0% [59.4% - 72.6%]	95.4% [92.5% - 98.3%]	NA
Mono-5	10,878	99.6% [99.5% - 99.7%]	91.9% [91.4% - 92.5%]	99.4% [99.2% - 99.5%]	NA
Mono-6	34,772	98.5% [98.3% - 98.6%]	78.9% [78.4% - 79.3%]	96.8% [99.6% - 97.0%]	NA
DC-1	7,383	84.7% [83.9% - 85.6%]	58.9% [57.8% - 60.0%]	0.1% [0.0% - 0.1%]	NA
DC-2	192	79.7% [74.0% - 85.4%]	57.3% [50.3% - 64.3%]	9.9% [5.7% - 14.1%]	NA
DC-3	164	87.2% [82.1% - 92.3%]	86.0% [80.7% - 91.3%]	2.4% [0.1% - 4.8%]	NA

Supplementary Table S3. Immune cell classification accuracy of ImmunIC, Garnett[14], Cell BLAST[15], and CellAssign[16]. CellAssign does not assign NK cells, monocytes, and dendritic cells as a separate population.

Pathways	Coverage (%)	P	Differentially Expressed Genes
EIF2 Signaling	14.3	< 0.001	RPL10, RPL11, RPL12, RPL13A, RPL14, RPL18, RPL22, RPL24, RPL28, RPL29, RPL30, RPL31, RPL32, RPL34, RPL35, RPL37, RPL39, RPL41, RPL8, RPLP2, RPS12, RPS13, RPS14, RPS2, RPS21, RPS23, RPS24, RPS26, RPS27A, RPS28, RPS7, RPS8
Role of IL-17A in Psoriasis	14.3	< 0.001	S100A8, S100A9
TNFR2 Signaling	9.4	< 0.001	FOS, JUN, NFKBIA
B Cell Activating Factor Signaling	9.3	< 0.001	FOS, JUN, NFKBIA, TNFSF13B
Interferon Signaling	8.3	< 0.001	IFI6, IFITM1, IFITM3
IL-17A Signaling in Fibroblasts	7.9	< 0.001	FOS, JUN, NFKBIA
IL-17A Signaling in Gastric Cells	7.7	0.0034	FOS, JUN
Coronavirus Pathogenesis Pathway	7.4	< 0.001	FOS, JUN, NFKBIA, RPS12, RPS13, RPS14, RPS2, RPS21, RPS23, RPS24, RPS26, RPS27A, RPS28, RPS7, RPS8
Primary Immunodeficiency Signaling	7.1	< 0.001	IGHA1, IGHG1, IGHM, IGKC
April Mediated Signaling	7.1	< 0.001	FOS, JUN, NFKBIA
MIF Regulation of Innate Immunity	6.8	< 0.001	FOS, JUN, NFKBIA
Regulation of eIF4 and p70S6K Signaling	6.7	< 0.001	RPS12, RPS13, RPS14, RPS2, RPS21, RPS23, RPS24, RPS26, RPS27A, RPS28, RPS7, RPS8
iNOS Signaling	6.4	< 0.001	FOS, JUN, NFKBIA
4-1BB Signaling in T Lymphocytes	5.9	0.0059	JUN, NFKBIA
TNFR1 Signaling	5.8	< 0.001	FOS, JUN, NFKBIA
mTOR Signaling	5.7	< 0.001	RPS12, RPS13, RPS14, RPS2, RPS21, RPS23, RPS24, RPS26, RPS27A, RPS28, RPS7, RPS8
CD27 Signaling in Lymphocytes	5.3	< 0.001	FOS, JUN, NFKBIA
Toll-like Receptor Signaling	5.1	< 0.001	FOS, JUN, NFKBIA, RPS27A

Supplementary Table S4. Differential signaling pathways of macrophages in 48 COVID-19 severe-progression cases, compared to 20 healthy controls.

Pathways	Coverage (%)	p	Genes
Thyroid Hormone Biosynthesis	50.0	0.0022	CTSD
Pathogenesis of Multiple Sclerosis	22.2	< 0.001	CCL3, CCL4
Role of IL-17A in Psoriasis	21.4	< 0.001	CXCL8, S100A8, S100A9
Differential Regulation of Cytokine Production in Macrophages and T Helper Cells by IL-17A and IL-17F	16.7	< 0.001	CCL3, CCL4, IL1B
Differential Regulation of Cytokine Production in Intestinal Epithelial Cells by IL-17A and IL-17F	13.0	< 0.001	CCL3, CCL4, IL1B
Interferon Signaling	11.1	< 0.001	IFI6, IFITM1, IFITM3, ISG15
Role of Hypercytokinemia/hyperchemokinemia in the Pathogenesis of Influenza	7.0	< 0.001	AREG, CCL3, CCL4, CXCL8, IL1B, ISG15
Role of IL-17F in Allergic Inflammatory Airway Diseases	6.4	< 0.001	CCL4, CXCL8, IL1B
TNFR2 Signaling	6.3	< 0.001	NFKBIA, TNFAIP3
Airway Inflammation in Asthma	6.1	< 0.001	CXCL8, RNASE2
Granzyme A Signaling	5.3	0.0204	H1-10
Inflammasome pathway	5.0	0.0214	IL1B
Coronavirus Replication Pathway	4.4	0.0011	IFITM1, IFITM3
IL-23 Signaling Pathway	4.4	0.0011	IL1B, NFKBIA
IL-10 Signaling	4.2	< 0.001	IL1B, IL1R2, NFKBIA
TREM1 Signaling	3.9	< 0.001	CCL3, CXCL8, IL1B
Role of MAPK Signaling in Inhibiting the Pathogenesis of Influenza	3.9	< 0.001	CXCL8, IL1B, NFKBIA
Toll-like Receptor Signaling	3.9	< 0.001	IL1B, NFKBIA, TNFAIP3
TNFR1 Signaling	3.9	0.0014	NFKBIA, TNFAIP3
IL-17A Signaling in Gastric Cells	3.9	0.0282	CXCL8
Role of Cytokines in Mediating Communication between Immune Cells	3.7	0.0016	CXCL8, IL1B
Role of IL-17A in Arthritis	3.5	0.0018	CXCL8, NFKBIA
LXR/RXR Activation	3.3	< 0.001	CLU, IL1B, IL1R2, S100A8
Granulocyte Adhesion and Diapedesis	3.2	< 0.001	CCL3, CCL4, CXCL2, CXCL8, IL1B, IL1R2
IL-6 Signaling	3.1	< 0.001	CXCL8, IL1B, IL1R2, NFKBIA
Activation of IRF by Cytosolic Pattern Recognition Receptors	3.1	0.0023	ISG15, NFKBIA
Atherosclerosis Signaling	3.1	< 0.001	CLU, CXCL8, IL1B, S100A8

Supplementary Table S5. Differential functional pathways of monocytes in the severe progression group, compared to the healthy control group.

References

1 Newman, A. M. *et al.* Robust enumeration of cell subsets from tissue expression profiles. *Nat Methods* **12**, 453-457, doi:10.1038/nmeth.3337 (2015).

2 https://satijalab.org/seurat/articles/pbmc3k_tutorial.html.

3 Smith, S. L. *et al.* Diversity of peripheral blood human NK cells identified by single-cell RNA sequencing. *Blood Adv* **4**, 1388-1406, doi:10.1182/bloodadvances.2019000699 (2020).

4 Cano-Gamez, E. *et al.* Single-cell transcriptomics identifies an effectorness gradient shaping the response of CD4(+) T cells to cytokines. *Nature communications* **11**, 1801, doi:10.1038/s41467-020-15543-y (2020).

5 Brockmann, L. *et al.* Molecular and functional heterogeneity of IL-10-producing CD4(+) T cells. *Nature communications* **9**, 5457, doi:10.1038/s41467-018-07581-4 (2018).

6 Zheng, G. X. *et al.* Massively parallel digital transcriptional profiling of single cells. *Nature communications* **8**, 14049, doi:10.1038/ncomms14049 (2017).

7 Guo, X. *et al.* Global characterization of T cells in non-small-cell lung cancer by single-cell sequencing. *Nat Med* **24**, 978-985, doi:10.1038/s41591-018-0045-3 (2018).

8 Rasouli, J. *et al.* A distinct GM-CSF(+) T helper cell subset requires T-bet to adopt a TH1 phenotype and promote neuroinflammation. *Sci Immunol* **5**, doi:10.1126/sciimmunol.aba9953 (2020).

9 Povoleri, G. A. M. *et al.* Human retinoic acid-regulated CD161(+) regulatory T cells support wound repair in intestinal mucosa. *Nat Immunol* **19**, 1403-1414, doi:10.1038/s41590-018-0230-z (2018).

10 Li, N. *et al.* Memory CD4(+) T cells are generated in the human fetal intestine. *Nat Immunol* **20**, 301-312, doi:10.1038/s41590-018-0294-9 (2019).

11 Eberhardt, C. S. *et al.* Functional HPV-specific PD-1(+) stem-like CD8 T cells in head and neck cancer. *Nature* **597**, 279-284, doi:10.1038/s41586-021-03862-z (2021).

12 Gangaev, A. *et al.* Identification and characterization of a SARS-CoV-2 specific CD8(+) T cell response with immunodominant features. *Nature communications* **12**, 2593, doi:10.1038/s41467-021-22811-y (2021).

13 Pauken, K. E. *et al.* Single-cell analyses identify circulating anti-tumor CD8 T cells and markers for their enrichment. *J Exp Med* **218**, doi:10.1084/jem.20200920 (2021).

14 Pliner, H. A., Shendure, J. & Trapnell, C. Supervised classification enables rapid annotation of cell atlases. *Nat Methods* **16**, 983-986, doi:10.1038/s41592-019-0535-3 (2019).

15 Cao, Z. J., Wei, L., Lu, S., Yang, D. C. & Gao, G. Searching large-scale scRNA-seq databases via unbiased cell embedding with Cell BLAST. *Nature communications* **11**, 3458, doi:10.1038/s41467-020-17281-7 (2020).

16 Zhang, A. W. *et al.* Probabilistic cell-type assignment of single-cell RNA-seq for tumor microenvironment profiling. *Nat Methods* **16**, 1007-1015, doi:10.1038/s41592-019-0529-1 (2019).
